# Event and Comment

**Published:** 1922-03

**Authors:** 


### DENTAL REGISTER

THE MAGAZINE OF DENTISTRY

Vol. LXXVI MARCH, 1922 No. 3

### EVENT AND COMMENT

**The Next Move in Dental Education.** In view of the discussions that may have disturbed the peace of mind in some dental educational circles, one is inclined to believe that we in the near future are to witness some changes in the method of dental education. Just what these changes are likely to be it is rather difficult now to foresee. In recent years there has been a constant restlessness and tendency to expand the curriculum, both quantitatively and qualitatively. These extensions seem to have demanded an increase in time, and additions to the subjects taught. Some additions of subjects not formerly thought necessary for dental students have been added. Most of these additions have developed normally, because the art of practice has similarly been expanded. Consequently, more intricate and exacting technical demands account for some of the needs for more time to study and work out methods of practice. Our own technical art and the necessary associated sciences have not developed except as necessity has demanded or forced them in an abnormal development.

We are continually being urged by practitioners to enlarge our artistic endeavors, and necessarily that means increased teaching of all subjects. Many practitioners make great efforts to keep up-to-date with both technical and scientific advancements.

In such subjects as chemistry, physics and biology we are discovering that we have got to know why we do certain things, even technical things. What is more complicating, we find that it is necessary that we should not only know *how* but *why* we do or have to learn these things that we formerly learned only in a general or perfunctory manner. And so we find out that the people when they learn of a new matter which is of concern to them that they want it put into practice. Much in the same way students continually demand a chance to see and know things that it takes time and care to teach. The educational field naturally grows, and in most cases it develops more rapidly than the profession realizes or even the teachers can assimilate and utilize. At any rate, however this growth comes about, we know that the demand is here and it will not be put off for a convenient season. This insistent demand that we must know *how* and *why* creates a condition that may be very inconvenient and even irritating at times to teachers and college officials; it is a good and proper stimulus to growth and we must accept and work it out the best our minds and desires can devise.

The days of empiricism and dogmatism are fast passing away—and they should—in medical and dental education; and with these advancements we should strive to keep up with the spirit and advancement of all scientific knowledge that shall make us more artistic as well as scientific. It is only because of well-grounded and long-tested theories that we can expect to have a reliable method or system of instruction that will stand the crucial test of practice. The fact is we are giving more importance to the study of basic scientific facts in teaching, we are giving more time to all these fundamental principles. All thoughtful educators are now prepared to give more time to these fundamentals, regardless of expense in time. In dental education we must realize that thorough study and patient practice must go hand in hand in any process of educating the future dentist.

The present survey which the Educational Council and the Carnegie Foundation are now making has for its object the determination of the actual state of dentistry for a scientific foundation upon which the practitioner can best be trained for the welfare of the public. Ultimately it is expected that it will show what system of education will insure the best methods and become most practicable for a completed and uniform system of training. Let us hope that this research will evolve some scheme of education that is reasonable and practicable, and which will give us the necessary stimulus for the better development of educated and trained scientists and artisans needed for the practice of our profession.

